# Ectoine can enhance structural changes in DNA *in vitro*

**DOI:** 10.1038/s41598-017-07441-z

**Published:** 2017-08-03

**Authors:** S. Meyer, M.-A. Schröter, M. B. Hahn, T. Solomun, H. Sturm, H. J. Kunte

**Affiliations:** 1 Federal Institute for Materials Research and Testing, D-12205 Berlin, Germany; 2 Institute of Biochemistry and Biology, University of Potsdam, D-14476 Potsdam, Germany; 3 Institute of Experimental Physics, Free University Berlin, Department of Physics, D-14195 Berlin, Germany; 4 Technical University Berlin, D-10587 Berlin, Germany

## Abstract

Strand breaks and conformational changes of DNA have consequences for the physiological role of DNA. The natural protecting molecule ectoine is beneficial to entire bacterial cells and biomolecules such as proteins by mitigating detrimental effects of environmental stresses. It was postulated that ectoine-like molecules bind to negatively charged spheres that mimic DNA surfaces. We investigated the effect of ectoine on DNA and whether ectoine is able to protect DNA from damages caused by ultraviolet radiation (UV-A). In order to determine different isoforms of DNA, agarose gel electrophoresis and atomic force microscopy experiments were carried out with plasmid pUC19 DNA. Our quantitative results revealed that a prolonged incubation of DNA with ectoine leads to an increase in transitions from supercoiled (undamaged) to open circular (single-strand break) conformation at pH 6.6. The effect is pH dependent and no significant changes were observed at physiological pH of 7.5. After UV-A irradiation in ectoine solution, changes in DNA conformation were even more pronounced and this effect was pH dependent. We hypothesize that ectoine is attracted to the negatively charge surface of DNA at lower pH and therefore fails to act as a stabilizing agent for DNA in our *in vitro* experiments.

## Introduction

In order to maintain an osmotic equilibrium with the surrounding medium, many bacteria synthesize and accumulate ectoine (1,4,5,6-tetrahydro-2-methyl-4-pyrimidinecarboxylic acid) as their main organic osmolyte^1–3^. For ectoine and other organic osmolytes the name compatible solutes was coined^4^, because they do not disturb the cell’s metabolism including nucleic acid and lipid metabolism, even at high molar cytoplasmic concentrations. Ectoine is beneficial for bacterial cells not only as a compatible solute, but also as a protectant of proteins mitigating the detrimental effects of freezing and thawing, drying and high temperatures^5^. The stabilizing effect of ectoine is explained by the *preferential exclusion* model^6^. According to the model, which has been proven true by the work of Oesterhelt and coworkers^7^, ectoine is excluded from the surface of proteins and their first hydration shell. The protein structure is stabilized by altering the strength of the hydrogen bonds between ectoine and water molecules in the surrounding^7–10^. Two distinct H-bond effects are responsible for the preferential exclusion of ectoine from the surfaces of proteins and the stabilizing effect of ectoine, namely: i) Favorable enthalpy change from ordering the water molecules by stronger water-ectoine H-bonds; ii) favorable entropy through the weakening of water-water H-bonding in the vicinity of the solute^7^.

However, according to the work of Smiatek *et al*. this mechanism seems not to apply to negatively charged surfaces. Their theoretical work provided data that indicate a *preferential binding* of the ectoine-derivative hydroxyectoine (5-hydroxy-1,4,5,6-tetrahydro-2-methyl-4-pyrimidine-carboxylic acid) to negatively charged spheres of biomolecules. In addition to the beneficial characteristics of ectoine that can be explained by preferential exclusion, some recent studies have found that ectoine can also protect entire eukaryotic cells from damages caused by cytotoxins or UV-A radiation^11, 12^. All these properties make ectoine a valuable compound and thus ectoine is used as the main component in many medical devices and cosmetics products. However, any details on how ectoine interacts with DNA are still unknown^13^. It was found that ectoine is able to reduce the melting temperature of double-stranded DNA depending on the GC-content of the nucleotide sequence and ectoine is therefore used as an enhancing agent for DNA amplification by polymerase chain reaction (PCR)^14^.

Since ectoine is influencing the DNA melting temperature and can protect entire cells against UV radiation, we investigated whether ectoine stabilizes DNA during storage and helps to protect DNA against ultraviolet (UV) radiation in a cell-free environment. UV light is divided in UV-A (400–320 nm, 3.1–3.9 eV), UV-B (320–290 nm, 3.9–4.3 eV) and UV-C (290–200 nm, 4.3–6.2 eV)^15^. The research on biological effects of UV radiation can look back on decades of studies and has revealed directly and indirectly damage to DNA molecules. It has been reported that direct DNA damage such as strand breakages within the sugar-phosphate backbone or nucleobase abstraction is carried out after absorption of UV-photons with energies from >6 eV^16, 17^. Apart from that, UV-B absorption leads to nucleobase modifications like (6-4) photoproducts (>4.2 eV)^18^ and cyclobutane pyrimidine dimers (CPDs, >3.8 eV)^18^, which can result in lesions within the DNA causing single or double strand breaks^19, 20^. The energetically lower UV-A light represents the lion’s share with 95% of the UV-light reaching the earth’s surface^21^. It is the major type of UV radiation to which living beings and their molecules are exposed, since all UV-C and approximately 90% of UV-B radiation is absorbed by the atmosphere^22^. Moreover, UV-A light is barely absorbed by atmosphere but is absorbed by lower dermal layers of human skin^21^. Therefore, UV-A can lead to DNA damage through indirect sensibilization processes of surrounding molecules^15^. Recent studies^20^ have additionally described the potential of damage during exposure by UV-A photons with energies of 3.4 eV (1 MJ/m^2^, 45 mW/cm^2^). This energy is sufficient to directly generate photoproducts like CPDs at the DNA and leads to DNA strand breaks. Thus, the general assumption according to which UV-A photons always need photosensitizers to transfer their energy to DNA was disproved^20, 23^.

DNA conformation, which may vary from supercoiled to relaxed forms, strongly regulates the access to the genetic information and has important consequences for the expression of genes^24, 25^. The morphology of plasmid DNA is very sensitive to environmental stresses. Even one single-strand break will lead to relaxation of supercoiled plasmid DNA resulting in the open circular conformation. Double-strand breaks will result in linear plasmids.

Accordingly, the subject of this study was to clarify whether ectoine helps to protect DNA in a cell-free *in vitro* setting. We investigated the effect of ectoine on DNA, exemplified for plasmid pUC19 DNA in pure water to exclude the interference of ions. Plasmid DNA was incubated in aqueous solution with and without ectoine and also exposed to UV radiation. We found out that ectoine enhances changes in DNA structure in a pH-dependent manner. We hypothesize that ectoine is possibly by being attracted to the negatively charged surface of the DNA polymer especially at low, non-physiological pH and therefore can not act as a stabilizing agent.

## Results

### Structural changes to plasmid DNA after incubation with ectoine

**Figure 1:**
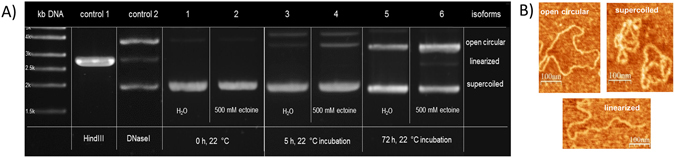
Representative images of pUC19 structures analyzed by gel electrophoresis and confirmed by AFM. (**A**) Electrophoretic mobility of pUC19 isoforms in 1% agarose gel: control (1) *Hin*dIII endonuclease digested pUC19, which results in linearized isoform after one double strand break; control (2) pUC19 unspecifically digested with DNaseI, which leads to open circular and linearized plasmid isoforms; (1–6) pUC19 under specific conditions (H_2_O or 500 mM ectoine solution at 22 °C) and incubation times are as indicated in the figure. (**B**) Isoforms of pUC19 confirmed by AFM:supercoiled (undamaged), open circular (single-strand break), linear (double-strand break).

**Figure 2:**
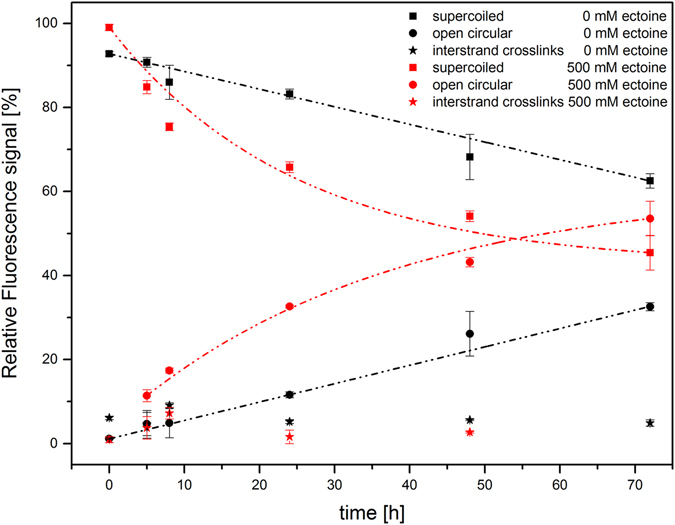
Plasmid pUC19 incubated for 0 h to 72 h with and without ectoine. Quantification of plasmid isoforms per sample (n = 3) via relative fluorescence signals from gel data. Incubation data without ectoine and in 500 mM ectoine solution show a decrease of the supercoiled form depending on the time and an increase of the open circular form. Amounts of interstrand crosslinks of plasmids are not changed. Lines are guides to the eye.

Ectoine is able to protect the native structure of proteins and lipid bilayers as well as mitochondrial DNA^26^. In order to investigate whether ectoine influences DNA structure, plasmid pUC19 in water was first subjected to 500 mM ectoine at a pH of 6.6. After incubation for 0 h, 5 h, 8 h, 24 h, 48 h, and 72 h at 22 °C, the samples were analyzed by agarose gel electrophoresis (Fig. 1) in order to visualize possible changes in the topology of the plasmid. Already after 5 h of incubation in ectoine solution, open circular isoforms of pUC19 were found indicating a change to the DNA backbone. In contrast, after 5 h in water without ectoine, pUC19 remained almost intact and mainly the unchanged supercoiled isoform was detected. After 72 h, the relaxed open circular (OC) isoform became also visible in the water sample. However, the water sample still contained less open circular DNA compared to the ectoine sample (see Fig. 2). We have identified and assigned the different isoforms of pUC19 DNA with the help of agarose gel electrophoresis. In addition, the attribution of the different isoforms was corroborated by Tapping Mode atomic force microscopy (TM-AFM). We isolated each isoform from the agarose gel and the AFM images of the isolated DNA, shown in Fig. 1B, confirmed the typical isoforms.

*In vivo*, relaxation of supercoiled DNA is catalyzed by isomerase enzymes (topoisomerase), which temporarily introduce strand breaks to DNA. Damage of DNA by shear force (*in vitr*o) or radiation leads to single-strand breaks and finally relaxation to OC DNA as well. The results obtained by AFM and gel electrophoresis imply that ectoine enhances strand breaks in DNA. To verify these results, pUC19 DNA was incubated with ectoine and treated with T7 exonuclease. Treatment with T7 exonuclease is a reliable method to detect DNA termini or gaps and nicks of double-stranded DNA^27, 28^. OC isoforms of pUC19 that arose after incubation with ectoine were degraded by T7 exonuclease, while supercoiled DNA was not altered. These findings confirmed that the observed changes in DNA conformation resulted from strand breaks during incubation with ectoine (Supplementary Figure S1).

### Quantification of changes in DNA structure

We quantified the GelRed-stained DNA isoforms from agarose gel and plotted the relative fluorescence signal of each plasmid isoform against the incubation time (Fig. 2). With pUC19 DNA in pure water, the supercoiled structure is decreasing over a period of 72 h and approximately 30% of pUC19 was converted into the OC isoform. In contrast, approximately 50% of the supercoiled structure of pUC19 decreased and converted into the OC isoform when incubated in ectoine solution (see also Table 1).

**Table 1:** Quantitative results from gel electrophoresis and atomic force microscopy outlining the formation of different pUC19 isoforms during incubation with ectoine.

pUC19 in 500 mM ectoine	supercoiled structure			open circular structure		
	5 h incubation	24 h incubation	72 h incubation	5 h incubation	24 h incubation	72 h incubation
AFM data: Mean % (STD), n = 9	84.4 (3.8)	68.0 (3.9)	47.0 (9.2)	15.3 (4.1)	31.0 (3.8)	50.7 (8.5)
Gel data: Mean % (STD), n = 3	84.8 (1.6)	65.8 (1.3)	45.4 (4.1)	11.4 (1.4)	32.6 (0.3)	53.6 (4.1)

*One-way ANOVA, all corrected p-values < 0.01.

**Figure 3:**
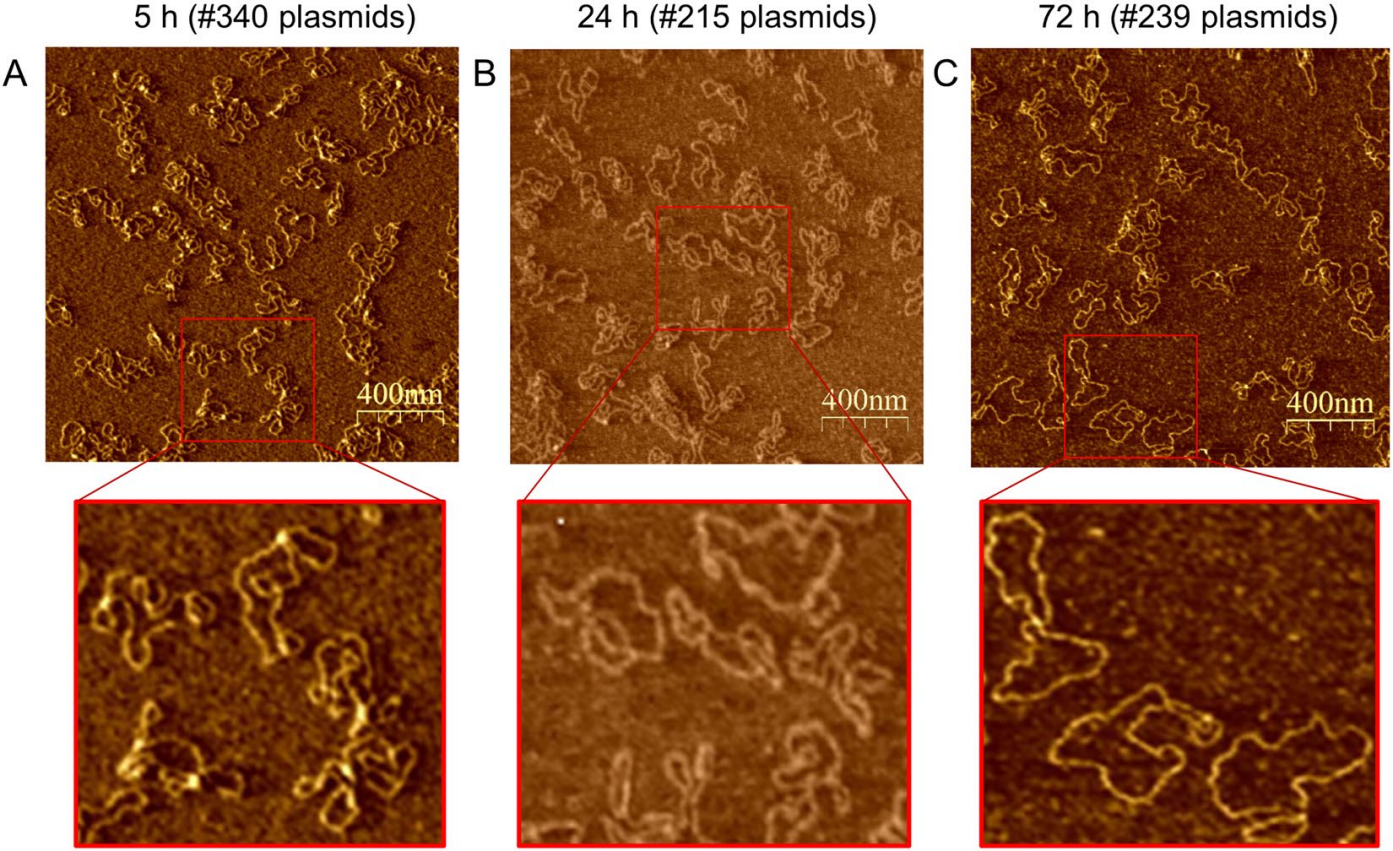
Representative AFM-amplitude images (1.9 µm scan) of pUC19. Plasmid pUC19 was incubated in aqueous ectoine solution (500 mM) at 22 °C for 5 h (**A**), 24 h (**B**) and 72 h (**C**). For AFM imaging at room temperature, pUC19 DNA was chemically fixed on ultra-smooth mica, which has been pre-silanized with APTES.

Quantification was repeated by amplitude controlled Tapping Mode AFM (TM-AFM), which is a standard technique to image susceptible biomolecules without detriment for the sample^29–31^. We incubated pUC19 with ectoine for 5 h, 24 h and 72 h and prepared samples from each incubation time for AFM imaging. From each sample, we took images from three different sites on the surface. In sum, 9 AFM amplitude images were taken and Fig. 3 shows three images from ectoine-treated pUC19 DNA as an example.

The images provide an insight into the structural diversity of pUC19 and the changes in structure that occur during incubation in aqueous ectoine solution. Statistical analyses of the results obtained by AFM showed a significant change in the amount of supercoiled plasmids that can be found after 5 h, 24 h and 72 h incubation, respectively (Table 1). The supercoiled plasmid diminished with increasing incubation time, while the open circular isoform is increased. AFM results are in good agreement with the data obtained by gel electrophoresis. Data from incubation times were always significant (corrected p-value from One-way ANOVA < 0.01) according to statistical analysis.

### Changes to DNA by UV-A irradiation in aqueous solution with ectoine

**Figure 4:**
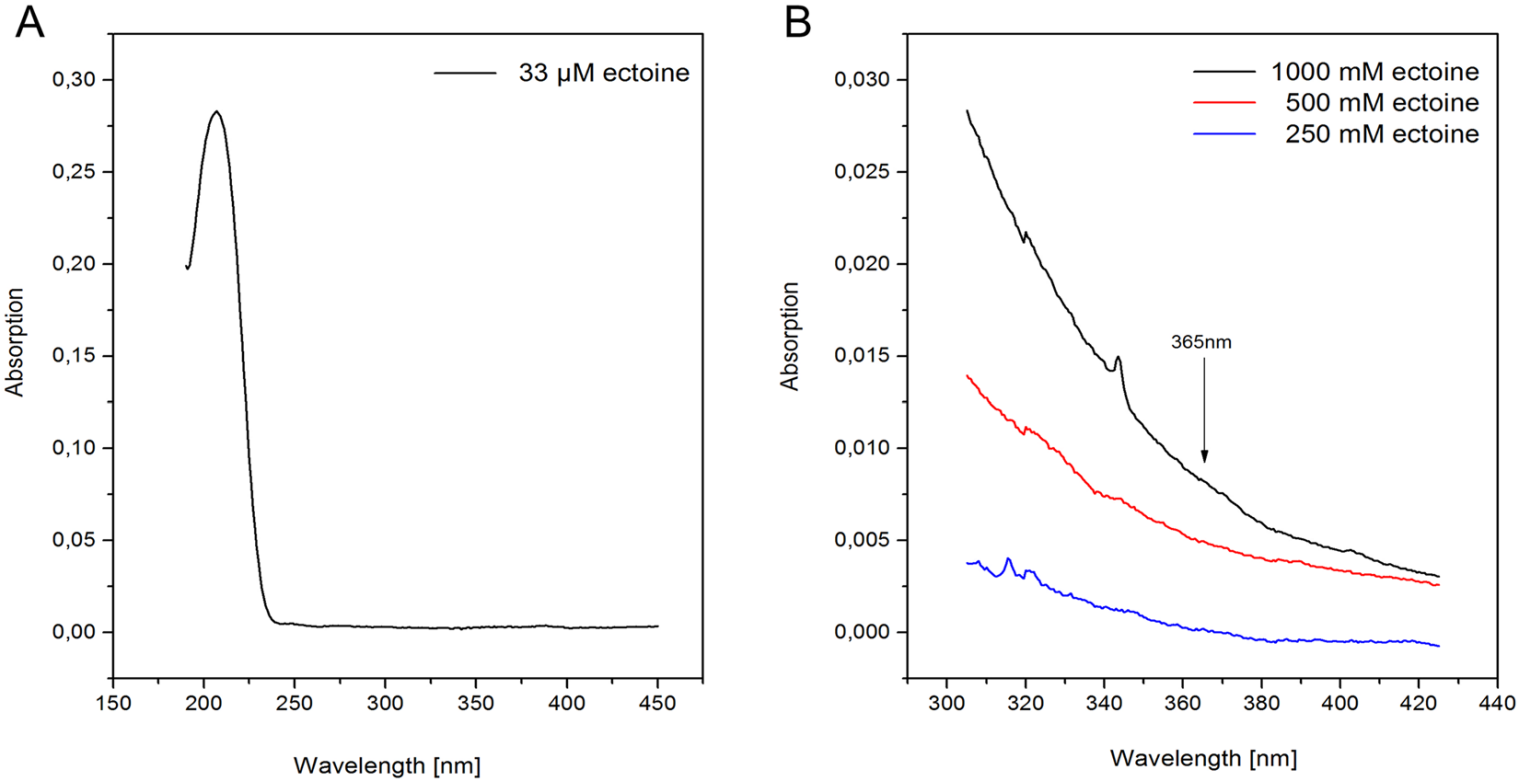
UV-absorption of ectoine in water. Absorption spectra of 33 µM ectoine (**A**) in the range of 190–450 nm and (**B**) at higher concentrations of ectoine (1000 mM, 500 mM, 250 mM) in the range of 305–425 nm.

In order to study the effect of UV-A light (3.4 eV, 365 nm, Supplementary Table S1) on plasmid DNA in ectoine solution (500 mM), structural changes of pUC19 were again analyzed by gel electrophoresis and AFM. The UV-spectrum of ectoine depicts a strong absorption (absolute extremum) at 207 nm (Fig. 4A). Ectoine still absorbs light at around 365 nm, which is visible at concentrations of 500 mM and 1000 mM.

**Figure 5:**
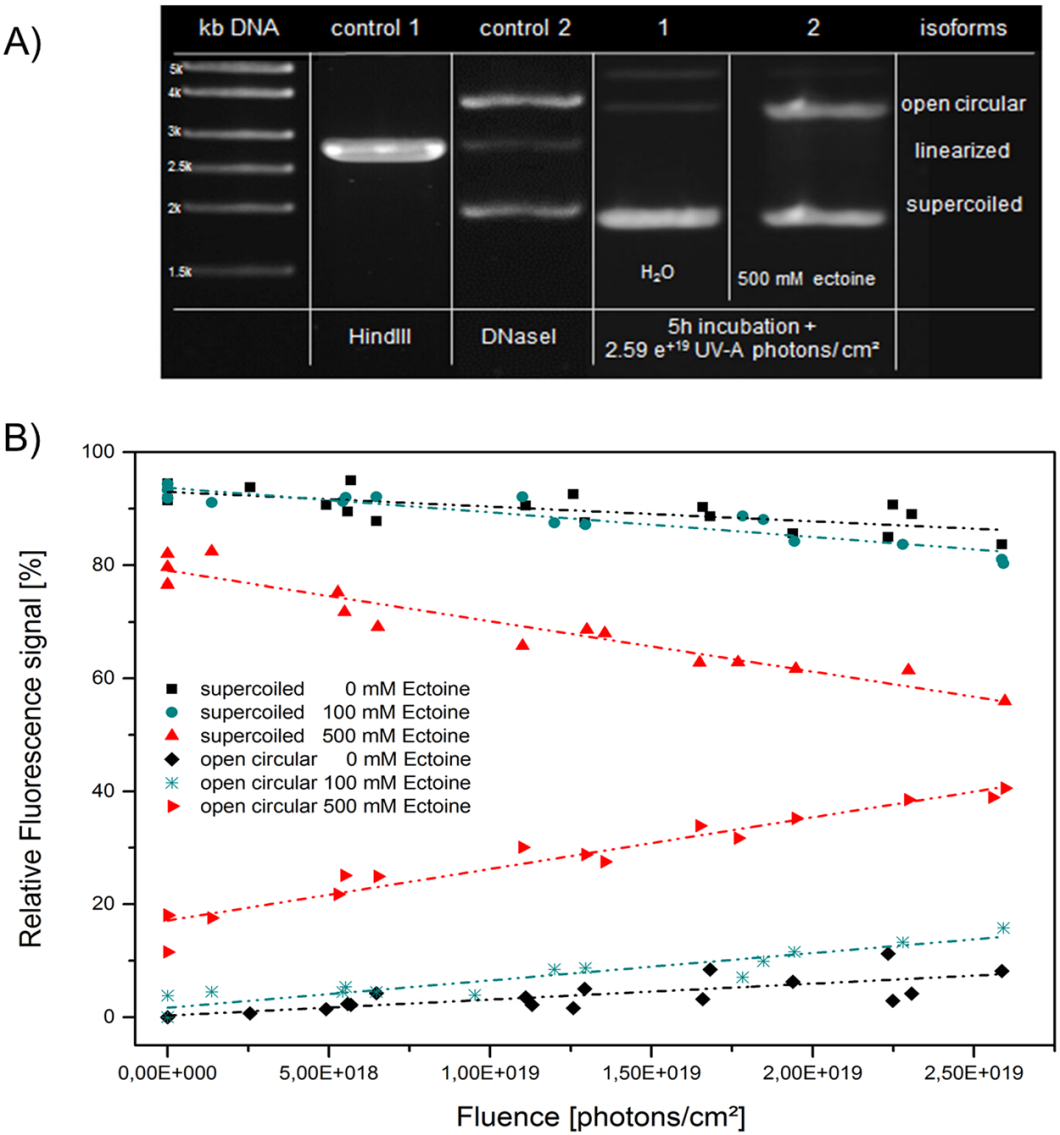
pUC19 samples incubated for 5 h and irradiated with UV-A (3.4 eV) with different photons per cm² in water and ectoine solution. (**A**) Electrophoretic mobility of pUC19 isoforms in 1% agarose gel: control (1) linearized pUC19 DNA after double-strand break by *Hin*dIII endonuclease; control (2) open circular and linearized pUC19 DNA after unspecific digest with DNaseI; (1) pUC19 in water after UV-A irradiation, (2) pUC19 in 500 mM ectoine solution after UV-A irradiation. (**B**) Quantification of plasmid isoforms per sample via relative fluorescence signal from gel data. Black data show samples without ectoine, turquois data show samples with 100 mM ectoine. Samples in 500 mM ectoine solution show a decrease of the supercoiled form with increasing amount of the open circular conformation. The lines are guides to the eye.

DNA of pUC19 was irradiated with UV-A light during 5 h incubation in aqueous solution. The irradiated DNA was separated by gel electrophoresis and the relative fluorescence signal of the stained DNA isoforms was plotted as a function of photon fluence per cm² (Fig. 5, Supplementary Table S1). Apparently, the impact of low energy UV-A light on DNA in water can be neglected. In contrast, DNA in ectoine solution was strongly affected by UV radiation. During irradiation the amount of native supercoiled plasmid diminished and correspondingly led to the formation of OC DNA (Fig. 5B). The severity of changes, which occurred during UV irradiation with ectoine, depend on the concentration of ectoine and a low concentration of 100 mM ectoine, for instance, had no effect.

Similar results were obtained by applying quantitative AFM (for details see Supplementary Table S2). As evidenced by T7 exonuclease experiments, the formation of OC DNA during UV irradiation resulted from strand breaks as well (Supplementary Figure S2).

### Influence of hydrogen ion activity (pH) on DNA structure during UV radiation and ectoine incubation

The UV irradiation and ectoine incubation experiments with pUC19 were carried out at a pH of 6.6, which was defined by the solution. To estimate how much the hydrogen ion concentration has influenced the structural changes of pUC19, the same irradiation and ectoine incubation experiments were repeated in Tris buffer at pH 7.5. It has to be stressed that the buffered solution at a pH of 7.5 resembles more the condition in the cytoplasm of bacteria compared to the ion free setting in pure water having a pH of 6.6^32^.

**Figure 6:**
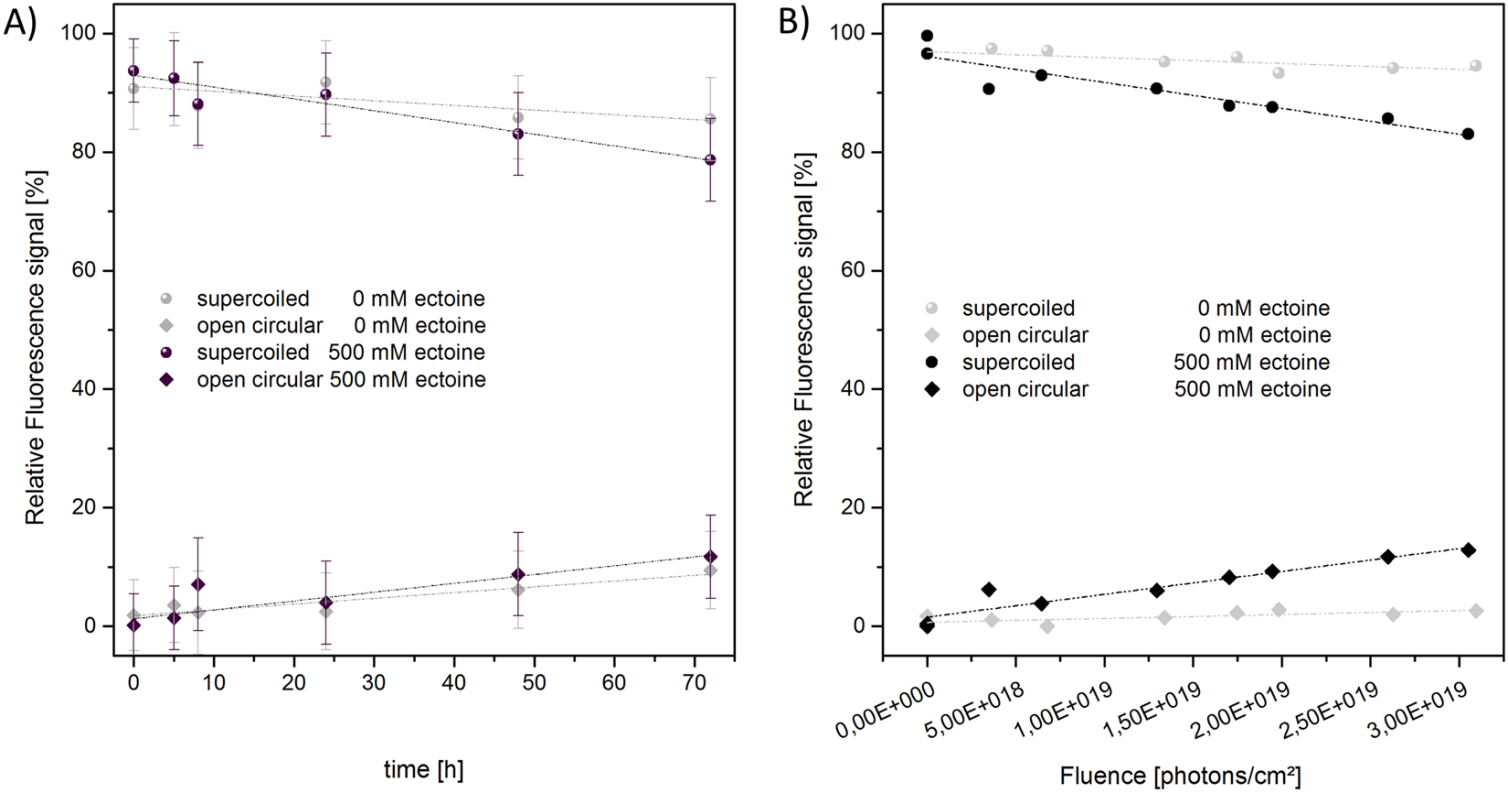
Stability of pUC19 in 10 mM Tris with and without ectoine. The change of plasmid configuration is shown in dependence of time and fluence: (**A**) pUC19 incubated for 0 h to 72 h without irradiation. In buffered solution, stability of DNA is unaltered in solution with ectoine compared to ectoine-free buffer. (**B**) pUC19 samples incubated for 5 h and UV-A irradiation. The lines are guides to the eye.

At a lower hydrogen ion activity of pH 7.5, ectoine did not cause any significant changes to DNA structure during a 72 h incubation (Fig. 6). Similarly, after UV radiation in Tris buffered ectoine solution less pUC19 plasmid was damaged (Fig. 6B) compared to the same irradiation experiment at pH 6.6 (Fig. 5).

## Discussion

By applying gel electrophoresis and Tapping Mode AFM, we were able to show for the first time that ectoine enhances the change of DNA structure. Gel electrophoresis and TM-AFM have proven strong and reliable tools in analyzing the different isoforms of plasmid DNA. With the help of these two completely different methods it was possible to differentiate linear, open circular and supercoiled DNA and quantified the different types of DNA with small error rates.

Double-stranded plasmid DNA is stored in a supercoiled, higher energy state, which is subjected to torsional stress to compact the long molecule. In contrast, the relaxed open circle form can arise after energy loss through insertion of a single strand break or by partial denaturation, which leads to an altered writhe^25, 33^. In the present study, it was shown that structural changes of supercoiled plasmids become apparent through an increasing amount of the open circular (OC) isoform. The data presented here revealed that a prolonged incubation in the presence of ectoine in a cell- and ion-free aqueous solution caused significantly more structural changes to DNA compared to incubation in water alone. The finding that ectoine is involved in changing the structure of DNA, most likely by enhancing strand breaks, came surprising to us since ectoine is known to protect biomolecules from denaturing, including the protection of DNA against e.g. UV stress in eukaryotic cell culture investigation^12^.

However, it has to be stressed that the findings presented here were obtained in an *in vitro* setting and might only have relevance for bacteria, which accumulate ectoine inside the cell in response to osmotic stress. The ectoine content in bacterial cells from the marine environment reaches approximately 500 mM but ectoine is amassed to higher concentrations in the cytoplasm depending on the osmolarity of the surrounding. This too was the reason to test ectoine at a concentration ranging from 100 mM to 1000 mM. Interestingly, ectoine has never been found in mammalian cells and it seems to be excluded from the cytoplasm and nucleus, respectively, of these cells. Experiments with human epithelia cells from the lung and keratinocytes have shown that ectoine is not transported inside the cell and even in permeabilized epithelia cells it is only transiently detectable (personal communication A. Bielstein, R&D bitop AG). Based on these results, a direct influence on the DNA of human cells, in all probability, can be ruled out. This is the more so if considered that ectoine as a protectant is applied to human cells at concentrations of 100 mM and below, which is too low a concentration to cause damage to DNA in the experimental setting applied in this study.

The influence of ectoine on biomolecules could be explained by a model, which postulates a possible exclusion or an attraction of this compatible solutes^9^. On the one hand, the model confirms the well-known preferential exclusion of compatible solutes from positive or neutral spheres. On the other hand however, it reveals a potential preferential binding of the ectoine-derivative hydroxyectoine to spheres with negatively charged surfaces as they can be found on DNA polymers. Bound to the sphere, it was calculated that hydroxyectoine is located approximately 0.6 nm above the surface. This is in good agreement with the position of the first water shell that surrounds biomolecules such as DNA and would result in a replacement of local water molecules. The position of water, however, depends on the ion concentration and estimations by other groups place the first hydration shell in closer vicinity to the surface with a distance of approximately 0.2 nm to 0.5 nm^32, 34–36^. From this it is assumed that the attraction of ectoine to the negatively charged DNA surface can be described as an interaction between a partly negative charge at the phosphate backbone and zwitterionic ectoine as dipole. The interaction is partly screened by water molecules and cations, when present at the phosphate backbone.

Based on the structure similarities, it is fair enough to assume that ectoine resembles hydroxyectoine in terms of its binding properties to surfaces. In support of this notion, calculations and Raman experiments have shown that ectoine strongly influences the water structure within the first hydration shell and binds around seven water molecules due to strong hydrogen bonds^10, 37, 38^. This results in a stabile ectoine-water complex in the surrounding of biomolecules^39^.

At first, it was assumed that the mechanism by which ectoine alters the structure of DNA lies in the fact that ectoine replaces water in the first hydration shell. Loss of water on DNA affects the transition from B-DNA to A-DNA^40^ and results in mitigated base pair interactions within DNA double strands^41, 42^. This could lead to a DNA molecule with stretches of single strands and remaining double strands with an altered writhe. Such amolecule would resemble plasmid DNA of the open circle (OC) conformation. Apart from that, relaxation of supercoiled DNA can be achieved by the insertion of single-strand breaks. *In vivo* relaxation of DNA is catalyzed by topoisomerases, which temporarily introduce strand breaks in DNA. Shear force (*in vitro*) or radiation leads to single-strand breaks and finally relaxation to OC DNA as well. With the help of the enzyme T7 exonuclease, strand breaks can be attacked *in vitro* and nucleotides from gaps and nicks of double-stranded DNA are removed. The experiments with T7 exonuclease revealed that all OC DNA that has been found after incubation with ectoine and UV irradiation, respectively, was generated by single-strand breaks^27, 28^.

Still, it is not clear, how ectoine is causing damage to plasmid DNA. Lindahl and coworkers have already demonstrated that plasmid DNA is reduced in stability by depurination and β-elimination processes in aqueous solution near neutral pH leading to cleavage of the phosphodiester backbone^43–47^. Ectoine-water in the vicinity of DNA could possibly lead to an increase of H^+^ ions, which facilitates depurination and β-elimination and consequently causes cleavage of the phosphodiester backbone. We were unable to detect any changes in pH in the presence of ectoine (error range pH +/− 0.4). However, our incubation experiments with Tris buffered aqueous solution (10 mM Tris) at pH 7.5 containing ectoine revealed that pUC19 showed less damage compared to incubation in non-buffered ectoine solution at pH 6.6 (Fig. 6). Based on these findings, we hypothesize that ectoine possibly facilitates damaging DNA by locally changing the pH.

Simultaneously incubating with ectoine and irradiating with UV-A (parameter in Supplementary Table S1) caused even more pronounced detrimental effects on DNA as shown in Fig. 5 compared to Fig. 2. Interestingly, the impact of UV-A alone on DNA was neglectable and the degree of damage during UV irradiation was dependent on the concentration of ectoine alone.

Accordingly, previous work by other groups has shown that UV-A photons alone are not able to cleave the sugar-phosphate backbone of DNA directly or to abstract nucleobases^16, 17^. Vogel and coworkers demonstrated that photons are required of at least UV-C light in the range of 6.50 to 8.94 eV to cause single strand breakages^17^. The study of Jiang^20^ showed the potential of UV-A photons with energies of 3.4 eV to directly generate photoproducts like cyclobutane pyrimidine dimers (CPDs) and finally causing DNA strand breaks^21, 23^. However, Jiang applied a much higher dose of 1 MJ/m² compared to our studies (3.4 eV, 0.14 MJ/m^2^).

The mechanism by which UV radiation and ectoine increase the damage to DNA remains unclear. Temperature and pH measurements of samples before and after irradiation showed no changes (error range +/− 0.5 °C and pH +/− 0.4). However, the damage to DNA was reduced at pH 7.5 in Tris buffered solution (Fig. 6). Only 10% of the supercoiled DNA was transformed into OC DNA while in unbuffered solution at pH 6.6 twice as much DNA was damaged. The results could be possibly explained by the additional formation of radicals in water^48, 49^. Secondary processes of this kind are well described in which photosensitization occurs by chromophores attached to DNA^48^. Our irradiation experiments were carried out applying UV light at 365 nm (3.4 eV), which is not absorbed by DNA^50^. The results presented here show that ectoine exhibits a maximum absorption in the UV-C range at 207 nm. At higher concentrations of 250 mM to 1000 mM, ectoine still absorbs light at around 365 nm (Fig. 4). Therefore, it is not unlikely that ectoine in the vicinity of DNA acts as photosensitizer by transferring its energy to the surrounding water or directly to DNA and thereby causing the damage.

## Conclusion

Here we report on the structural changes to DNA that are enhanced by the osmoprotectant ectoine at high concentrations of 500 mM in a cell-free artificial system. Based on our data we conclude that ectoine decreases the stability of supercoiled plasmid DNA at 500 mM through enhancing the insertion of single-strand breaks, which leads to the formation of energetically favorable relaxed open circle plasmid. This effect was only observed at pH below the cytoplasmic hydrogen activity of 6.6 and after prolonged incubation of pUC19 DNA in aqueous solution containing ectoine. The effect under these conditions was even more pronounced in combination with UV-A irradiation. Interestingly, in Tris buffer roughly above the neutral pH, which resembles more the condition in the cytoplasm of bacteria, incubation with ectoine had no effect on DNA. Similarly, the damages caused by UV light in the presence of ectoine at pH 7.5 were mitigated. This is in agreement with the observation *in vivo*, where ectoine accumulates up to 20% of the dry cell mass of halophilic bacteria and does not cause any damage to the bacterial DNA. The mechanism by which ectoine influences DNA stability is not clear. The formation of structured water by ectoine in the vicinity of DNA could possibly lead to this effect. In further studies, the mode of action of ectoine on DNA has to be investigated in detail. In particular, it has to be clarified whether ectoine possibly protect DNA against other stressors. Ongoing research in our laboratory indicates that ectoine might be potent protector for DNA against other types of radiation. Most importantly, further research has to be carried out to help us understanding on if and how ectoine acts on DNA *in vivo*.

## Methods

### Plasmid DNA preparation

The plasmid pUC19 (2686 base pairs) was transformed in *Escherichia coli* Top10 (One Shot® TOP10 Competent Cells, Invitrogen) by using the recommended standard protocol from Invitrogen. After cultivating the cells, the plasmids were isolated and purified with NucleoBond® Xtra Midi Plus (Macherey-Nagel) and resuspended in ultra-pure water (conductance 0.055 µS cm^−1^, pH 6.6) or in 10 mM Tris buffer (pH 7.5). The concentration and purity of plasmids was determined by using NanoDrop2000c (Thermo Scientific).

Deoxyribonuclease I (DNase I) is an endonuclease that digests unspecifically double- and single-stranded DNA. To prepare a plasmid control which consists of open circular and linearized conformations, the incubation of 1 µg pUC19 in 0.01 Units DNase I (Sigma) for 10 min at 37 °C was carried out before analyzing by gel electrophoresis.

### Incubation of plasmids in ectoine

For determining the plasmid change, solutions of the plasmid sample at concentrations of 25 ng/μl were adjusted in ultra-pure water (conductance 0.055 µS cm^−1^, pH 6.6) or in 10 mM Tris buffer (pH 7.5). The plasmid solutions, in ultra-pure water or ectoine (1,4,5,6-tetrahydro-2-methyl-4-pyrimidinecarboxylic acid, purity ≥99% (HPLC), bitop AG, Germany) solution (500 mM, pH 6.6), were incubated for 10 min to 3 days at 22 °C (+/−1 °C). Using gel electrophoresis and atomic force microscopy, the plasmid samples in ectoine were compared to plasmid samples without ectoine.

### T7-exonuclease experiment

T7 exonuclease experiments were carried out to clarify the mechanism of structural change in plasmids by removing nucleotides from 5′termini or gaps and nicks of double-stranded DNA. In this approach 4 Units T7 Exonuclease (Biolabs) per 1 µg DNA were incubated for 30 min at 37 °C. The digested DNA samples were analyzed by gel electrophoresis.

### UV-A irradiation

Experiments were performed using 200 µl plasmid solution (25 ng/µl) in UV microcuvettes (Brand). The UV lamp (Hamasutsu, Lightningcure^TM^ LC8) was operated with additional two filters providing an average value of P = 1.95 mW/cm^2^ in UV-A range (365 +/− 5 nm). We performed the irradiation of pUC19 samples with 0 to ~2.6 ·10^+19^ photons/cm² (max. ~14 J/cm²). Each 365 nm-photon has an energy of 3.4 eV. The irradiances are determined using High Resolution Spectrometer (HR 2000+, Ocean Optics Inc.). Three biological replicates of each plasmid sample, pUC19 in water or Tris buffer, with and without ectoine, were irradiated 4 times for 30 min within 5 h incubation time. At each irradiation break we collected samples for gel electrophoresis. In addition to that, temperature measurements were carried out before and after each irradiation.

### UV-absorption spectra of ectoine

The UV data were obtained from a dual-trace UV-Vis spectrophotometer (SPECORD 210, Analytik Jena) using quartz glass cuvettes (Suprasil, Heraeus) with a layer thickness of 10 mm. After calibration (ultra-pure water: conductance 0.055 µS cm^−1^, pH 6.6), the UV absorption spectra of ectoine-aqueous solution (33 µM) in the range of 190–450 nm and additionally ectoine solution (1 M, 0.5 M, 0.25 M) in the range of 305–425 nm was measured. During the measurements, the scan speed was fixed at 0.5 nm/s. For data analysis WinASPECT software (Version: 2.3.1.0) was used.

### Agarose gel electrophoresis

Gel electrophoresis was performed to identify different plasmid structures and to determine topological changes after incubation and UV-A irradiation of plasmids in ectoine. Each channel of 1% agarose gel was run with 75 ng plasmids at 50 V for 90 minutes in gel electrophoresis. The DNA was stained with GelRed^I^ (GeneON GmbH) and visualized as well as quantified by measuring the fluorescence intensity of each plasmid structure (Herolab E.A.S.Y.® Doc plus, E.A.S.Y. Win-Software).

### AFM sample preparation

Atomic force microscopy was performed to determine the structure of DNA plasmids in ectoine-aqueous solution at different incubation conditions and after UV-A irradiation. The samples were prepared on mica, which is commonly used as ultra-smooth substrate for the deposition of biomolecules. We functionalized the surface based on a procedure^51^ by incubating an aliquot of 20 µl of 0.05% APTES solution ((3-Aminopropyl)-triethoxysilane) for one minute at room temperature on freshly cleaved mica and purged with ultra-pure water (conductance 0.055 µS cm^−1^). Nitrogen (Linde, 5.0) was used to blow-dry the functionalized mica plates. Under these conditions we are able to get an APTES layer, which is less than one nanometer thick. An aliquot of plasmid DNA (10 µl with 2 ng/µl in 1 mMol Tris-HCl buffer, pH 7.5) was deposited on the APTES treated mica, incubated for two minutes and purged at least three times with ultra-pure water. The samples were blow-dried and stored in a glass vessel with phosphorus pentoxide to reduce the humidity.

### Tapping Mode atomic force microscopy (TM-AFM)

Tapping Mode AFM was performed in air by using a Nanotec Electronica SL (Madrid, Spain) microscope. In order to receive high spatial resolution we used a cantilever with diamond-like carbon (DLC) whiskers at the tip (supplier: NT-MDT), which have a curvature radius of about 1–3 nm. The resonance frequency was between 90–160 kHz. Images were collected at a scan frequency of 1.356 Hz and the scan size was chosen to 2.5 µm since statistical purposes require a large number of plasmids on each AFM image, resulting in a pixel resolution of ~4.9 nm. Extreme care was required to adjust the feedback in a way that nor the DNA is destroyed neither the tip is contaminated. The relative humidity in the closed chamber was reduced, however the hygroscopic sample surfaces will still exhibit adsorbed and absorbed water, increasing tip-sample adhesion and energy dissipation. Thus, topography images appear still blurred, hence the amplitude signal is used for analysis. Since only conformation as well as contour length of the plasmids is analyzed, the information of the DNA height is not required.

### AFM image post-processing

For visualization, analysis and post-processing of the AFM images WsXM software (version 4.0 Beta 6.4)^52^ was used. Since the immobilization procedure causes artefacts like silan aggregates bright spots in the images were suppressed. If necessary, a post-processing in terms of a low-pass-filter was applied after the carefully performed background subtraction of artefacts caused by substrate or scanning process. In all cases the color scale was optimized for enhancing the plasmid contour.

### Data analysis and quantification

Analyzing gel electrophoresis data, plasmid structures of three biological replicates from incubation samples (incubated for 0 h, 5 h, 24 h, 72 h) and UV-A irradiated (365 +/− 5 nm, 0.14 MJ/m²) samples were quantified, respectively in ectoine solution (500 mM) and without ectoine. The mean value of incubated samples and data from UV-A experiment were used to compare these data with our results from AFM experiments. In AFM experiments three replicates per condition (plasmids incubated for 5 h, 24 h, 72 h and exposed to UV-A (365 +/− 5 nm, max 0.14 MJ/m²) with ectoine (500 mM) were deposited on mica and three different parts of each sample were imaged. At least, we generated 9 AFM-images per experimental condition to analyze and quantify the different plasmid structures. Here, we considered 340 (5 h, ectoine), 215 (24 h, ectoine), 239 (72 h, ectoine) plasmids for analysis. A visual assessment of each image was carried out by two independent persons. In this study, following criteria were used to count the plasmids: (1) Plasmid structures have to be completely visible on the image. (2) Plasmids have to be recognizable as single molecule and not lie on top of each other. (3) Differentiate between plasmid isoforms: supercoiled, open circular or linear structure. For statistical analysis of AFM data we have chosen the Anderson Darling test for confirming normal distribution and the One-Way ANOVA plus the Tukey Test for analysing variances of independent or correlated samples. We compared each group due to our criteria and the topological differences in plasmids. Here, the procentual amounts of each plasmid structure were taken as a basis, not the actually counted from every image. Results are expressed as the mean for each fraction.

## Electronic supplementary material

Supplementary information
