# Supplementary material for: Ectoine can enhance structural changes in DNA in vitro
Source: Sci Rep. 2017 Aug 3;7:7170. doi: 10.1038/s41598-017-07441-z (PMC5543045; doi:10.1038/s41598-017-07441-z)
Supplement: Supplementary file 1 — Supplementary information [file 41598_2017_7441_MOESM1_ESM.pdf]

Supplementary information

Ectoine can enhance structural changes in DNA *in vitro*

S. Meyer*[1,2], M.-A. Schröter[1], M. B. Hahn[1,3], T. Solomun[1], H. Sturm[1,4], H.-J. Kunte[1]

[1]Federal Institute for Materials Research and Testing, D-12205 Berlin, Germany
[2]Institute of Biochemistry and Biology, University of Potsdam, D-14476 Potsdam, Germany
[3]Institute of Experimental Physics, Free University Berlin, Department of Physics, D-14195 Berlin, Germany.
[4]Technical University Berlin, D-10587 Berlin, Germany

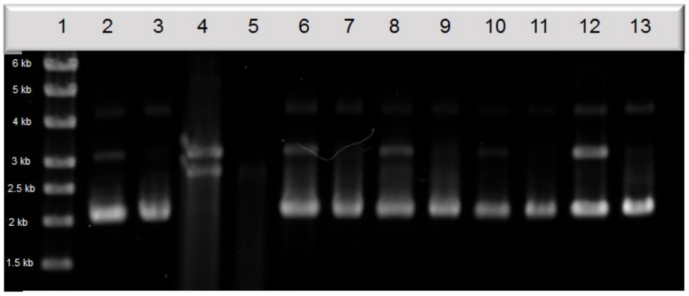

1) DNA Ladder
2) pUC19 control
3) pUC19 control T7 Exonuclease digested
4) pUC19 DNaseI digested
5) pUC19 DNaseI + T7 Exonuclease digested
6) pUC19 72 h incubated
7) pUC19 72 h incubated + T7 Exonuclease digested
8) pUC19 72 h incubated in ectoine
9) pUC19 72 h incubated in ectoine + T7 Exonuclease digested
10) pUC19 UV-A irradiated within 5 h incubation
11) pUC19 UV-A irradiated within 5 h incubation + T7 Exonuclease digested
12) pUC19 in ectoine UV-A irradiated within 5 h incubation
13) pUC19 in ectoine UV-A irradiated within 5 h incubation + T7 Exonuclease digested

Supplementary Figure S1| T7 Exonuclease experiment with selected samples.

Supplementary Table S1| UV irradiation parameter.

	**Irradiation time**			
	**30 min**	**60 min**	**90 min**	**120 min**
**Power per cm² of UV-lamp**	P = 1,95 mW/cm² ≙ 3,58 +/- 0,05* 10^{15} photons/s * cm²			
**Wave-length (UV-A)**	λ = 365 nm (+/- 5 nm)			
**Photon energy**	E = 3,40 +/-0,05 eV = 5,44 +/- 0,08 *10^{-19} J			
**Irradiated energy per area**	3,51 J/cm² = 3,51 * 10^{-2} MJ/m²	7,02 J/cm² = 7,02 * 10^{-2} MJ/m²	10,53 J/cm² = 10,53 * 10^{-2} MJ/m²	14,04 J/cm² = 14,04 * 10^{-2} MJ/m²
**Fluence of photons**	0,64 +/- 0,01* 10^{19} photons/cm²	1,29 +/- 0,02 * 10^{19} photons/cm²	1,93 +/- 0,03 * 10^{19} photons/cm²	2,58 +/- 0,03 * 10^{19} photons/cm²

Supplementary Table S2| Amount of isoforms from selected UV-A irradiated samples analyzed by gel electrophoresis and atomic force microscopy.

pUC19 in 500 mM Ectoine	supercoiled structure		open circular structure	
UV-A irradiated	without ectoine	500 mM ectoine	without ectoine	500 mM ectoine
AFM data: Mean % (STD), n=9	92.1 (0.5)	82.9 (2.1)	7.0 (0.5)	17.1 (2.1)
Gel data: %	86.3	56.0	6.2	40.6

Due to a lack of replicates a statistical analysis of gel data is needless.
